# Advances in fenugreek breeding: novel genetic and *omic* approaches

**DOI:** 10.3389/fpls.2025.1674013

**Published:** 2025-11-21

**Authors:** Solanki Bal, Amit Baran Sharangi, Mohd Saeed, Ali Alkhathami, Samra Siddiqui, Nadiyah M. Alabdallah

**Affiliations:** 1 School of Smart Agriculture, Adamas University, Kolkata, India; 2 Department of Plantation Spices Medicinal & Aromattic Crops, Bidhan Chandra Krishi Viswavidyalaya, Mohanpur, India; 3 Department of Biology, College of Science, University of Hail, Ha’il, Saudi Arabia; 4 Department of Clinical Laboratory Sciences, College of Applied Medical Sciences, King Khalid University, Abha, Saudi Arabia; 5 Department Health Services Management, College of Public Health and Health Informatics, University of Hail, Hail, Saudi Arabia; 6 Basic & Applied Scientific Research Centre, Imam Abdulrahman Bin Faisal University, Dammam, Saudi Arabia

**Keywords:** fenugreek, genetic diversity, biotechnology, omics, genomics

## Abstract

Fenugreek (*Trigonella foenum*-*graecum* L.) has long been valued for its diverse applications in culinary, medicinal, and cultural traditions across the globe. In recent years, this underutilized legume has attracted growing attention from researchers due to its rich profile of bioactive compounds and its potential in sustainable agriculture and health-based industries. Despite its historical significance and remarkable adaptability to different agro-climatic zones, fenugreek has not received adequate focus in mainstream breeding programs. However, recent developments in genetic improvement strategies, mutation breeding, molecular markers, and biotechnological techniques have started to unlock its untapped potential. This review brings together the latest advancements in fenugreek research, ranging from conventional breeding methods to cutting-edge molecular and omic approaches. Mutation breeding using physical and chemical mutagens like EMS and sodium azide has played a significant role in generating phenotypic variability and improving key traits such as yield, early maturity, and secondary metabolite concentration. Marker-assisted analysis using RAPD, AFLP, ISSR, and combined systems has contributed to mapping genetic diversity and identifying promising genotypes. Alongside these, the use of tissue culture techniques-including callus culture, cell suspension, protoplast regeneration, and organogenesis-has facilitated *in vitro* propagation and enhanced the synthesis of valuable phytochemicals like diosgenin and trigonelline. Furthermore, genetic transformation via *Agrobacterium rhizogenes* has enabled the development of hairy root cultures, which serve as efficient systems for bioactive compound production. The integration of omics technologies-genomics, transcriptomics, proteomics, and metabolomics-has offered new insights into the molecular basis of trait expression, metabolic pathways, and regulatory networks involved in fenugreek’s therapeutic potential. Notably, transcriptome studies have advanced our understanding of steroidal saponin biosynthesis, while metabolomic and proteomic tools have provided dynamic perspectives on plant physiology and compound accumulation under different conditions. Altogether, these advancements highlight a multidimensional approach to fenugreek improvement, paving the way for the development of elite cultivars with enhanced agronomic performance, higher nutritional and pharmaceutical value, and greater resilience to environmental stresses. This comprehensive overview underscores the need for continued investment in interdisciplinary research to fully harness the potential of fenugreek as both a functional food and a medicinal crop for the future.

## 1 Introduction

Plants have served humanity for centuries, not only as a vital source of nutrition but also as repositories of therapeutic compounds. Among the many botanical species with a rich history in both traditional medicine and culinary use, *Trigonella foenum*-*graecum* L., commonly known as fenugreek, stands out for its diverse and multifaceted roles (Chaudhary et al., 2018a). Widely recognized for its nutritional richness and pharmacological attributes, fenugreek has long been integrated into ancient healthcare systems such as Ayurveda, Chinese, and Tibetan medicine, where it was employed to treat ailments ranging from digestive issues to metabolic disorders (Al-Habori and Raman, 2002b; Acharya et al., 2006).

As a member of the Fabaceae family, fenugreek is an annual, dicotyledonous herb that displays substantial morphological diversity, making it an interesting subject for genetic and breeding studies (Randhawa et al., 2012). Variability in plant height, seed traits, and growth habit not only speaks to its adaptability across different agro-climatic regions but also offers significant opportunities for genetic improvement through breeding interventions (Bal et al., 2020, 2021, 2022).

The seeds of fenugreek are particularly valued for their bioactive constituents, which include alkaloids, flavonoids, saponins, and steroidal compounds (Jani and Thaker, 2009). These phytochemicals are responsible for fenugreek’s therapeutic applications in managing diabetes, inflammation, skin disorders, and gastrointestinal disturbances (Zandi et al., 2017a; Faizal et al., 2024b). In addition to its medicinal profile, fenugreek is also nutritionally rich-its leaves and seeds are dense in proteins, dietary fiber, vitamins, and essential minerals, making it a valuable crop for enhancing dietary quality (Zandi et al., 2017b).

Despite these versatile applications, fenugreek has been relatively underutilized in mainstream crop improvement programs (Toker et al., 2007b). One contributing factor is the limited global research focus on its breeding and genetic enhancement. Most traditional efforts have emphasized phenotypic selection and landrace utilization, with minimal exploitation of modern biotechnological tools (Amir et al., 2023; Yadav et al., 2007).

In recent decades, however, the landscape is rapidly changing with the integration of advanced breeding strategies, including mutation breeding, molecular marker-assisted selection, plant tissue culture, and transgenic approaches (Kapoor and Srivastav, 2010). Mutation breeding, using agents such as EMS, sodium azide, and gamma radiation, has generated promising phenotypic variants with improved traits such as early maturity, higher yield, and enhanced secondary metabolite production (Yadav et al., 2007; Toker et al., 2007b).

Simultaneously, molecular marker systems like RAPD, AFLP, and ISSR have been successfully applied to assess genetic diversity and construct linkage maps for breeding purposes (Sundaram and Purwar, 2011; Sharma et al., 2022; Patel et al., 2023b). These tools have significantly improved our understanding of fenugreek’s genetic structure and variability, enabling the identification of elite genotypes for targeted improvement (Choudhary et al., 2013b).

In addition to these, more recent marker systems such as Inter-Primer Binding Site (iPBS) and Single Nucleotide Polymorphisms (SNPs) are gaining traction in fenugreek genetic studies. iPBS markers, which are based on conserved retrotransposon sequences, have shown high polymorphism and reproducibility, making them useful in assessing genome-wide variability. Similarly, SNP markers, due to their abundance and stability, offer high-resolution insights into trait inheritance and are increasingly applied in association mapping and marker-assisted selection (Randhawa et al., 2012).

Moreover, plant tissue culture techniques, including callus induction, cell suspension cultures, and protoplast cultures, have facilitated *in vitro* regeneration and metabolite enhancement (Aasim et al., 2014a). Studies have shown increased diosgenin and trigonelline content under controlled conditions, highlighting the potential of biotechnological interventions in metabolite production (Ramesh et al., 2010; Mishra et al., 2021; Joshi and Handler, 1960).

Notably, the use of *Agrobacterium rhizogenes*-mediated transformation to develop hairy root cultures has yielded significant improvements in secondary metabolite synthesis, particularly for diosgenin and trigonelline (Merkli et al., 1997; Raheleh et al., 2011; Patel et al., 2023b).

Parallel to these approaches, the emergence of “omics” technologies-genomics, transcriptomics, proteomics, and metabolomics-has ushered in a new era of system-level understanding (Chaudhary et al., 2018a). These disciplines offer insights into gene expression, protein interactions, and metabolic fluxes, revealing complex biochemical and genetic pathways associated with fenugreek’s therapeutic potential (Agrawal et al., 2015; Gupta et al., 2019a).

Despite these encouraging developments, the full potential of fenugreek remains largely untapped due to gaps in integrative research and a lack of consolidated breeding strategies. Therefore, the present review aims to provide a holistic overview of the progress made in fenugreek breeding, with a focus on integrating traditional and modern genetic approaches, omics-based discoveries, and biotechnological innovations. This comprehensive account seeks to inform and inspire further research toward the development of high-yielding, nutritionally superior, and pharmacologically potent fenugreek cultivars suitable for diverse applications.

## 2 Legacy of fenugreek: from ancient healers to modern science

Fenugreek (*Trigonella foenum-graecum*) is a versatile herbaceous plant known for its adaptability and broad utility across culinary and medicinal domains (Singh et al., 2022). Native to the Mediterranean and extensively cultivated in South Asia, fenugreek has carved a niche in food systems globally. Its dried seeds—commonly known as methi dana—are a staple in Indian kitchens, while fresh tender shoots and young leaves are a seasonal delicacy in winter dishes such as methi paratha and saag (Faisal et al., 2024). Beyond South Asia, fenugreek seeds have long served as a flavoring agent in bread, cheese, and syrups, making them a cherished ingredient in global gastronomy.

Historically, fenugreek’s significance extended beyond the kitchen. Ancient Egyptians used it to ease childbirth, stimulate lactation, and in embalming practices. Hippocrates praised its soothing properties, and Romans used it in treatments for fevers, respiratory conditions, and wound healing. Traditional texts in Ayurveda, Chinese, Arabic, Greek, and Latin pharmacopoeias extensively document its medicinal virtues.

Scientific studies have validated many of these ethnobotanical claims. Fenugreek seed extracts, rich in galactomannan fiber and trigonelline, help regulate blood sugar (Mullaicharam et al., 2013b). Clinical trials show that 15–25 g of fenugreek seed powder daily significantly lowers fasting blood glucose and HbA1c levels in diabetic patients. Its seeds, containing flavonoids, saponins, and coumarins, also suppress pro-inflammatory mediators like TNF-α and IL-6, making fenugreek valuable in arthritis and asthma management (Faizal et al., 2024a). Saponins such as diosgenin reduce cholesterol absorption, while trigonelline and 4-hydroxyisoleucine improve lipid metabolism. Estrogenic compounds support lactation in postpartum women (Salehi Surmaghi, 2008).

Nevertheless, due caution is required in its clinical use, particularly regarding dosage and patient-specific conditions. As herbal remedies gain popularity, consultation with healthcare professionals remains essential for ensuring both safety and efficacy.

## 3 Botanical characteristics and taxonomic heritage of fenugreek

Fenugreek is an annual herb belonging to the Fabaceae family. The genus name *Trigonella* comes from the Greek “trigonon” (triangle), reflecting the triangular shape of its leaflets, while the species epithet *foenum-graecum* translates to “Greek hay,” denoting its use as fodder in ancient Greece (Montgomery, 2006).

The plant grows up to 60 cm tall with trifoliate leaves, serrated margins, and small flowers ranging from pale yellow to whitish-purple. Its pods, 3–11 cm long, contain 10–20 hard, yellowish-brown seeds that are rhomboidal and bitter (Visuvanathan et al., 2022).

Fenugreek shows substantial genetic variability in morphology and yield traits. Significant differences among genotypes in plant height, pod dimensions, and seed yield demonstrate its potential for selective breeding. Its seeds are also rich in galactomannans, flavonoids, coumarins, saponins, and alkaloids, contributing to therapeutic effects like glycemic control and anti-inflammation.

Agro-morphological characterization using UPOV descriptors has revealed variability in traits like days to flowering, pod curvature, seed color, and biomass accumulation. Such evaluations are crucial for trait-based selection, yield stability, and cultivar registration (Bal, 2023; Singh et al., 2022; Chaudhary et al., 2023; Sharma et al., 2021; Bhat et al., 2021; Sharangi et al., 2024).

## 4 Origins and global expansion of fenugreek

Fenugreek is believed to have originated in the eastern Mediterranean and North Africa, though some suggest domestication first occurred in Iran (Patel et al., 2021; Ahmed and Kumar, 2023). Today, it is cultivated widely in Egypt, Saudi Arabia, Pakistan, Algeria, China, India, Turkey, Ukraine, Spain, and Italy. Major exporters include India, Morocco, China, and Turkey (Singh et al., 2022).

India’s Rajasthan and Gujarat lead national production, with Rajasthan alone contributing nearly 80%. Arid and semi-arid zones favor seed development, oil yield, and metabolite content. Morocco and Turkey also show strong regional adaptation and yield variation.

Over 100 fenugreek varieties exist globally, reflecting centuries of human selection and ecological adaptation. Phenotypic studies in Iran, India, and Ethiopia show wide variability in seed traits, flowering time, and trigonelline content (Joshi and Handler, 1960). North African ecotypes tolerate saline soils, while Indian lines display drought resistance and early maturity.

Multi-environment trials confirm fenugreek’s stability under drought and salinity stress while retaining high diosgenin levels (Rahman et al., 2024). Global germplasm banks in Canada, India, and Australia conserve elite lines for future breeding.

## 5 Fenugreek’s germplasm repository

Fenugreek (*Trigonella foenum-graecum*), a member of the *Trigonella* genus, represents a vast genetic pool with approximately 260 documented species worldwide (Petropoulos, 2002a). This genus includes a diverse range of cultivable species such as *T. balansae*, *T. calliceras*, *T.* sp*icata*, *T. occulta*, *T. lilacina*, *T. corniculata*, *T.* sp*inosa*, *T. caerulea*, *T. radiata*, *T. maritima*, *T. cretica*, *T. polycerata*, and *T. foenum-graecum*. Among these, *T. foenum-graecum* is the most extensively cultivated due to its high demand in the pharmaceutical and nutraceutical industries, owing to its rich profile of bioactive compounds (Petropoulos, 2002a).

Fenugreek cultivation spans Asia, Africa, and Europe, with India, China, Türkiye, Pakistan, Morocco, Egypt, and Ethiopia being major producers (Acharya et al., 2008a). Turkey alone harbors 51 Trigonella species, reflecting its role as a genetic hotspot (Dangi et al., 2004).

Major germplasm repositories include the Plant Gene Resources of Canada, the National Bureau of Plant Genetic Resources (India), the University of Melbourne (Australia), and USDA ARS (Washington). These collections safeguard genetic diversity crucial for improving diosgenin yield, seed quality, and stress resilience (Petropoulos, 2002a; Basu, 2006a; Acharya et al., 2008b).

Conventional breeding of fenugreek faces challenges due to its self-pollination. Selective hybridization, induced mutations, and genetic recombination have been employed to overcome this (Bal et al., 2019, 2022; Prajapati et al., 2010; Basu et al., 2014; Rajoriya et al., 2016). Biotechnological tools—transformation, tissue culture, and marker-assisted selection—provide new avenues for targeted improvement and trait optimization.

## 6 Breeding approaches and the role of mutation in fenugreek improvement

Fenugreek (*Trigonella foenum-graecum* L.) exhibits a chromosomal count of 2n = 16 (Darlington and Wylie, 1945). Cytogenetic studies have revealed the presence of B-chromosomes (Raghuvanshi and Joshi, 1968) and multiple trisomic conditions (Singh and Singh, 1976), underscoring the genetic complexity that forms the foundation of fenugreek improvement (Martin et al., 2011b). Given its diverse commercial applications—ranging from diosgenin-rich seeds for steroidal industries to protein-, fiber-, and mucilage-rich seeds for nutritional and industrial purposes—breeding strategies have focused on enhancing both agronomic performance and metabolite yield (Petropoulos, 2002a; Acharya et al., 2008a; Amir et al., 2023).

Conventional methods such as selection and hybridization have been widely employed, though fenugreek’s predominantly self-pollinating nature limits recombination (Toker et al., 2007a). Classified as a rarely cross-pollinated species due to stigma receptivity preceding anther maturation, controlled pollination remains feasible during early floral stages, enabling targeted crosses (Allard, 1960; Petropoulos, 2002a; Basu, 2006b). Global germplasm collection and induced mutagenesis have therefore become cornerstones of fenugreek breeding (Petropoulos, 2002a).

Mutation breeding has emerged as a particularly powerful approach to broaden the genetic base and develop superior cultivars (Yadav et al., 2007; Toker et al., 2007b; Tiwari and Lavanya, 2012). Both spontaneous and induced mutations have contributed to traits such as increased diosgenin levels, higher yields, early maturity, and improved stress tolerance. Spontaneous mutants like RH 3129, derived from a Moroccan cultivar, displayed twin pods and elevated diosgenin content (Laxmi et al., 1980; Petropoulos, 2002a). Induced mutagenesis has employed physical and chemical agents including γ-rays, UV radiation, colchicine, sodium azide (NaN_3_), ethyl methanesulfonate (EMS), and methyl methanesulfonate (MMS), leading to mutants with altered growth habits, enhanced alkaloid content, and improved adaptability (Auerbach, 1961; Roy and Singh, 1968; Jain and Agarwal, 1987; Siddiqui et al., 2007). Colchicine treatments induced tetraploidy with improved economic traits (Roy and Singh, 1968), while EMS produced determinate mutants with altered growth, pest resistance, and novel cytogenetic profiles (Chaudhary and Singh, 2001; Kapoor and Srivastav, 2010).

Comparisons of mutagenic agents have revealed differential efficiencies. Combined low-dose treatments of EMS, MMS, and NaN_3_ increased steroidal sapogenin content, whereas higher concentrations caused reductions (Brain and Williams, 1983b; Jain and Agarwal, 1987). Ethidium bromide proved more effective than UV radiation for mutagenesis (Gadge et al., 2012). Similarly, EMS was reported to be more effective than sodium azide or γ-rays, though higher doses led to reduced germination, stunted seedling growth, and lower survival (Bashir et al., 2013a; Rajoriya et al., 2016; Naaz et al., 2023). Low doses of γ-rays (100 Gy) and EMS (0.2%) were beneficial for certain traits, while higher doses (200 Gy and 0.4% EMS) significantly enhanced trigonelline content (Yadav et al., 2021; Khorrami et al., 2024).

Practical outcomes of mutation breeding include the development of early-maturing and determinate growth habit mutants, ensuring synchronized maturity in short growing seasons and higher productivity (Basu et al., 2008). Induced mutations have also produced fenugreek lines with improved drought tolerance, validated using multi-trait genotype–ideotype distance index (MGIDI) analysis (Jyothsna et al., 2024).

Recent molecular tools have expanded the scope of mutation breeding. Start Codon Targeted (SCoT) markers have been used to characterize mutation-induced diversity (Khorrami et al., 2024), while *in vitro* applications integrated with mutagenesis have accelerated cultivar development.

A notable milestone was the release of ‘Tristar’, the first fodder cultivar in Western Canada, developed by Agriculture and Agri-Food Canada in 2004, derived from the Iranian accession L3314 (PI-138687) (Basu, 2006a). Collectively, these advances highlight the synergistic role of cytogenetics, induced mutagenesis, and modern molecular tools in diversifying the fenugreek gene pool and developing cultivars with improved yield, resilience, and nutraceutical value (Rahman et al., 2024).

## 7 Comparative advantages and limitations of fenugreek in breeding and utilization

Fenugreek (*Trigonella foenum-graecum* L.) offers several advantages that make it a promising crop, yet it also presents notable limitations that require careful consideration. Agronomically, fenugreek is highly adaptable to arid and semi-arid conditions, with low input requirements, nitrogen-fixing ability, and a short life cycle, making it suitable for sustainable agriculture and marginal soils (Petropoulos, 2002a; Acharya et al., 2006). At the same time, it is constrained by relatively low seed yield compared to other legumes and susceptibility to diseases such as powdery mildew and root rot, while its predominantly self-pollinating nature restricts genetic recombination and narrows the breeding base (Singh et al., 2022). From a breeding perspective, the species exhibits significant genetic diversity across landraces and ecotypes, and both spontaneous and induced mutations have generated genotypes with improved diosgenin content, yield, and stress tolerance (Basu, 2006a; Yadav et al., 2007; Toker et al., 2007b). However, stable *in vitro* regeneration remains difficult to achieve, and the application of advanced omics-based tools lags behind that of major legume crops (Chaudhary et al., 2018; Gupta et al., 2019c). Industrially, fenugreek seeds are rich in bioactive compounds such as diosgenin, galactomannan, trigonelline, and flavonoids, which support their widespread use in pharmaceuticals, nutraceuticals, and functional foods (Faizal et al., 2024a). Nonetheless, challenges remain in the form of bitter taste and strong odor, limited consumer acceptability in some markets, and difficulties in standardizing bioactive content. When comparing morphological and molecular approaches, classical morphological and yield studies remain cost-effective and easily measurable, with UPOV descriptors offering standardized trait characterization (Singh et al., 2022; Sharma et al., 2021). Yet, these traits are highly influenced by the environment, while molecular markers such as RAPD, AFLP, ISSR, SNPs, and iPBS provide more precise insights into genetic variation and marker–trait associations, albeit requiring greater technical expertise and infrastructure (Patel et al., 2023a). Taken together, fenugreek exemplifies a crop of high potential, but its future improvement depends on integrating its agronomic adaptability, industrial value, and molecular insights while addressing yield, disease susceptibility, and breeding challenges.

## 8 Molecular markers in assessing genetic diversity in fenugreek

### 8.1 RAPD and AFLP based approach

Random Amplified Polymorphic DNA (RAPD) and Amplified Fragment Length Polymorphism (AFLP) markers have been extensively employed to evaluate genetic diversity in fenugreek (*Trigonella foenum-graecum* L.), a leguminous crop known for its medicinal, nutritional, and agronomic significance (Patel et al., 2023). RAPD markers, in particular, have been favored for their simplicity, cost-effectiveness, and efficiency over other marker systems such as AFLP, ISSR, or their combinations (Sundaram and Purwar, 2011; Choudhary et al., 2013a). Sundaram and Purwar (2011) analyzed 61 accessions across two related Trigonella species using 18 RAPD primers, yielding 141 bands with 52.85% polymorphism and genetic similarity scores ranging from 0.66 to 0.90. Choudhary et al. (2013) reported 57.66% polymorphism across 17 varieties, emphasizing the mismatch between morphological and molecular data. Haliem and Al-Huqail (2014) extended RAPD studies to wild fenugreek accessions, observing polymorphism rates exceeding 90% for both molecular and biochemical markers. Modi et al. (2016) found 82.50% polymorphism using 11 RAPD primers among five cultivars, and Mamatha et al. (2017) used 30 RAPD markers to classify 48 genotypes into 10 clusters, with a polymorphism range from 50.00% to 91.66%. Korat et al. (2018) highlighted the need for multi-marker approaches by observing variability in clustering patterns using RAPD and ISSR. Recent integrations of RAPD with high-throughput sequencing have refined diversity analysis and clustering resolution. On the other hand, AFLP markers have demonstrated strong discriminatory power in assessing fenugreek diversity, offering wide genome coverage and high reproducibility (Patel et al., 2023c). Kumar et al. (2012) used 17 fluorescently labeled AFLP primer combinations to detect 669 peaks across multiple genotypes, highlighting close genetic relationships among select varieties. Recorded 1852 polymorphic loci in 24 accessions from Oman, Pakistan, and Iraq, with the highest diversity observed in Omani landraces. Detected 147 polymorphic bands among 20 Iranian landraces, with similarity values ranging from 44% to 94%. Gupta et al. (2019d) used a combination of AFLP and ISSR markers to examine 30 accessions, reporting 81.6% polymorphism and similarity coefficients between 0.42 and 0.88. Sharma et al. (2021) revealed clear genetic differentiation between wild and cultivated populations using 12 AFLP primers, while Patel et al. (2023c) reported 67.5% polymorphism in Indian landraces and recommended integrating molecular data with morphological traits for better resolution. Collectively, both RAPD and AFLP approaches have proven instrumental in revealing the genetic complexity of fenugreek (Kumar et al., 2012). While RAPD provides a quick and cost-effective assessment of diversity, AFLP offers deeper insights into population structure and intra-species variability (Spandan and Pooja, 2019). Moving forward, combining these markers with high-throughput genomic techniques such as SNP genotyping, ISSR, and next-generation sequencing (e.g., GBS) is expected to enhance fenugreek breeding programs, facilitate marker-assisted selection, and ensure more efficient germplasm conservation.

### 8.2 ISSR based approach

Inter Simple Sequence Repeat (ISSR) markers have been increasingly recognized for their efficiency in assessing genetic diversity, yet their application in fenugreek (*Trigonella foenum-graecum* L.) remains relatively underexplored. To date, ISSR-based fenugreek studies are limited, with only a few in-depth investigations providing valuable insights into the genetic variability of this medicinal and culinary herb.

One of the pioneering studies was conducted by Randhawa et al. (2012), who analyzed 49 fenugreek accessions sourced from diverse geographic locations. Their comprehensive approach integrated nineteen morphometric traits along with eighty-six ISSR markers to examine genetic relationships. Out of an initial set of 100 ISSR primers, twenty-one were polymorphic, yielding a total of 186 amplicons with an impressive 92.4% polymorphism rate, indicating significant genetic variation. Cluster analysis based on ISSR data revealed that 47 accessions formed a major genetic group, displaying an overall similarity of approximately 65%. Additionally, morphometric data reinforced this clustering pattern, confirming the correlation between genetic distance and phenotypic traits.

In another study, Mamatha et al. (2017) focused on 48 fenugreek genotypes to uncover genetic variation using ISSR markers. After screening twenty primers, they identified ten primers that exhibited strong amplification, producing a total of 48 polymorphic bands, with an average of 4.8 bands per primer. The resulting dendrogram showcased ten distinct clusters, with a genetic similarity coefficient ranging from 0.59 to 1.00 and a mean similarity coefficient of 0.75. These findings underscored the genetic complexity and evolutionary divergence among fenugreek accessions (Bal et al., 2022).

Recent advancements have further expanded the scope of ISSR applications in fenugreek. Patel et al. (2022) employed ISSR and SSR markers in combination to assess the genetic diversity of fenugreek landraces across India. Their study revealed a higher resolution of polymorphic loci (87%) than previous reports, demonstrating that ISSR markers as a robust tool for population structure analysis. Similarly, Gupta et al. (2020) examined 55 fenugreek accessions from distinct agro-climatic regions, reporting a polymorphism information content (PIC) ranging from 0.71 to 0.92, confirming the markers’ high discriminatory power.

Moreover, Sharma et al. (2023) introduced ISSR-based genetic fingerprinting techniques for fenugreek, employing fluorescent ISSR markers, which improved fragment detection accuracy. Their findings not only validated previous studies but also suggested potential applications in fenugreek breeding programs aimed at trait selection and conservation of elite genotypes.

These studies collectively establish ISSR markers as a potent molecular tool for assessing fenugreek genetic diversity. As research continues, integrating ISSR markers with other molecular techniques such as SNP and AFLP markers may provide a more holistic genetic landscape, facilitating improved breeding strategies and conservation efforts for fenugreek germplasm.

### 8.3 iPBS approach

Inter-Primer Binding Site (iPBS) markers are a relatively novel class of retrotransposon-based molecular markers that utilize the conserved tRNA-binding sites of long terminal repeat (LTR) retrotransposons to amplify genomic regions. These markers are highly reproducible, informative, and do not require prior sequence information, making them particularly useful in orphan or under-studied crops like *Trigonella foenum-graecum* (Kalendar et al., 2010; Kalendar and Schulman, 2006).

Although iPBS markers have not yet been widely applied in fenugreek, their successful deployment in closely related legumes—such as *Medicago truncatula*, *Pisum sativum*, and *Cicer arietinum*—suggests significant promise for assessing genetic diversity, detecting insertional polymorphisms, and mapping retrotransposon activity in the fenugreek genome (Hajyzadeh et al., 2015; Ghareeb et al., 2022).

In a comparative analysis of marker systems for legumes, iPBS was found to exhibit higher polymorphism rates than RAPD and ISSR, often exceeding 85% polymorphic loci in tested accessions. This high degree of polymorphism is particularly advantageous in fenugreek, which possesses extensive intra-specific variation across global landraces and ecotypes. The reproducibility and simplicity of iPBS protocols further enhance their suitability for genetic fingerprinting and evolutionary studies (Baránek et al., 2012).

Recent fenugreek research directions have shown an inclination toward integrating high-resolution markers like iPBS and SNPs to better dissect population structure and marker-trait associations. For instance, ongoing studies are exploring the utility of iPBS primers to develop DNA fingerprints for Indian and Mediterranean fenugreek germplasm. Early data from pilot projects have revealed amplification patterns yielding 6–12 loci per primer, with polymorphism information content (PIC) values ranging from 0.62 to 0.91, indicating their strong discriminatory power (Jdeed et al., 2023; Patel et al., 2023b).

Moreover, the epigenetic and genome-wide mobility of retrotransposons targeted by iPBS markers makes them ideal for stress-response studies, which is particularly relevant in fenugreek, given its adaptation to arid, saline, and nutrient-poor soils. The combination of iPBS with phenotypic and metabolomic data is likely to enhance the understanding of genotype-environment interactions, thereby guiding future breeding efforts aimed at resilience and nutritional enhancement.

iPBS markers represent a promising addition to the molecular toolbox for fenugreek. Their ability to uncover genome-wide polymorphism without prior genomic data, along with their adaptability across lab settings, positions them as a viable option for both population genetics and breeding applications. As fenugreek research embraces next-generation molecular tools, iPBS systems are expected to complement SNP genotyping and transcriptomic approaches in the quest to develop elite, climate-resilient, and pharmacologically enriched cultivars.

### 8.4 SNP approach

Single Nucleotide Polymorphisms (SNPs) represent one of the most abundant and informative classes of molecular markers in plant genomics. These markers are defined by single base-pair variations at specific loci within the genome and are widely distributed across both coding and non-coding regions. Due to their high density, co-dominant inheritance, and amenability to high-throughput genotyping, SNPs have become indispensable in genomic selection, association mapping, and marker-assisted breeding.

In the context of *Trigonella foenum-graecum*, SNPs are increasingly being explored to dissect genetic diversity, identify quantitative trait loci (QTLs), and enhance selection efficiency. Although comprehensive SNP panels are still in development for fenugreek, initial transcriptome and genotyping-by-sequencing (GBS) studies have successfully identified thousands of SNPs distributed across metabolic and stress-response pathways (Gupta et al., 2019c; Chaudhary et al., 2018b). These SNPs offer high-resolution insight into allele frequencies among diverse accessions, aiding in the understanding of population structure and evolutionary dynamics.

For instance, Gupta et al. (2019a) reported over 3,800 putative SNPs through RNA-Seq-based transcriptomic analysis of fenugreek, particularly in genes associated with diosgenin biosynthesis and abiotic stress responses. These SNPs were used to annotate candidate genes involved in steroidal saponin production, thus opening avenues for trait-linked marker development. Similarly, ongoing GBS-based efforts in Indian and North African germplasm pools are generating SNP datasets for genome-wide association studies (GWAS) to identify loci controlling yield, flowering time, and metabolite concentration.

SNP markers have also demonstrated their utility in genebank curation and core collection development. Using SNP genotyping, researchers are now able to establish distinct genetic clusters within fenugreek germplasm collections, supporting the identification of redundant accessions and guiding parental selection in breeding programs (Jdeed et al., 2023). Moreover, their co-dominant nature makes SNPs ideal for heterozygosity estimation, an essential parameter in hybrid seed production and purity testing.

When integrated with phenotypic, metabolomic, and transcriptomic data, SNP markers provide a comprehensive platform for genomic prediction models that can significantly accelerate trait-based selection. With decreasing genotyping costs and expanding genomic resources, the future of fenugreek improvement will likely hinge on SNP-based technologies integrated into breeding pipelines.

SNP markers offer unparalleled resolution and power in fenugreek genetics. Their ability to uncover subtle allelic variations, combined with compatibility with modern sequencing platforms, positions SNPs as a cornerstone of next-generation fenugreek breeding programs aimed at enhancing productivity, quality, and stress resilience.

### 8.5 Targeted improvement of hidden traits in fenugreek through molecular interventions

To effectively unlock the hidden genetic and biochemical potential of fenugreek (*Trigonella foenum-graecum* L.), it is essential to adopt a comprehensive strategy that merges traditional plant breeding with contemporary molecular and biotechnological innovations (Zhou et al., 2019). The foundation of this approach lies in an in-depth characterization of the available germplasm pool using a combination of morphological traits, biochemical markers, and molecular tools. This step is crucial for identifying underutilized accessions that possess traits of strategic interest—such as high diosgenin content, salinity tolerance, delayed flowering, or enhanced phytochemical profiles (Choudhary et al., 2013b; Sundaram and Purwar, 2011). Once diversity is established, mutation breeding using chemical mutagens like ethyl methane sulphonate (EMS) or physical agents such as gamma irradiation can be applied to induce new genetic variation (Modi et al., 2016). These induced mutants are then rigorously screened for improved agronomic performance or metabolite enrichment. In parallel, plant tissue culture systems—including callus induction, organogenesis, and suspension cultures—can support the rapid propagation and optimization of elite or mutant lines while enhancing metabolite biosynthesis under controlled conditions (Mamatha et al., 2017).

To further enhance the expression and yield of pharmaceutically valuable compounds such as trigonelline, saponins, and flavonoids, genetic transformation techniques, especially Agrobacterium rhizogenes-mediated hairy root culture, are employed to generate genetically stable and high-yielding root biomass (Sharma et al., 2022). In recent years, the integration of multi-omics platforms—namely genomics, transcriptomics, and metabolomics—has opened up unprecedented possibilities for dissecting complex metabolic pathways and identifying candidate genes involved in the biosynthesis and regulation of critical secondary metabolites, such as those linked to the diosgenin biosynthetic route (Patel et al., 2023b). Once these genetic targets are identified, advanced molecular breeding techniques like SNP genotyping, genotyping-by-sequencing (GBS), and genome-wide association studies (GWAS) are applied to map quantitative trait loci (QTLs), enabling precision breeding and trait pyramiding (Gupta et al., 2019b).

Moreover, for large-scale application and industrial use, bioreactor-based cultivation systems combined with elicitors such as methyl jasmonate or salicylic acid have been shown to significantly upregulate secondary metabolite production by mimicking stress-induced biosynthetic pathways. With the advent of genome editing technologies, especially the CRISPR/Cas system, there is a growing need to refine protoplast isolation and plant regeneration protocols specific to fenugreek, which remains a technical bottleneck but holds transformative potential. Ultimately, by merging these diverse but complementary approaches—including omics data, environmental adaptability, and trait stability—it is possible to construct a resilient and scalable breeding pipeline. This pipeline would not only address agronomic productivity but also target enhanced nutritional and therapeutic value, paving the way for next-generation fenugreek cultivars suited to both farming and functional food industries.

### 8.6 Critical insights, emerging perspectives, and research gaps in fenugreek improvement

Fenugreek (*Trigonella foenum-graecum* L.) has emerged as a promising multipurpose crop due to its rich reservoir of bioactive compounds such as diosgenin, trigonelline, saponins, and flavonoids, which exhibit significant pharmacological effects including anti-diabetic, anti-inflammatory, and cholesterol-lowering properties (Zandi et al., 2017a; Faizal et al., 2024a). Its adaptability across diverse agro-climatic zones, especially saline and arid regions, underscores its potential in climate-resilient agriculture (Bal et al., 2021; Rahman et al., 2024). Extensive germplasm collections preserved in global and national gene banks—including landraces and wild relatives—provide valuable genetic resources for future improvement (Petropoulos, 2002a; Acharya et al., 2008a). Traditional breeding strategies have been enhanced by mutation breeding using agents like EMS and gamma radiation, which have led to the development of novel genotypes such as RH 3129 with elevated diosgenin content (Kapoor and Srivastav, 2010; Yadav et al., 2021). Molecular markers including RAPD, ISSR, AFLP, iPBS, and SNPs have been widely employed to assess genetic diversity, revealing significant intra- and inter-specific variation that can be harnessed for trait improvement (Sundaram and Purwar, 2011; Gupta et al., 2019d; Sharma et al., 2022). Parallel advancements in omics—particularly transcriptomics, proteomics, and metabolomics—have begun to uncover the complex biosynthetic pathways and regulatory mechanisms responsible for key phytochemicals, such as those involved in the diosgenin biosynthesis pathway (Vaidya et al., 2013; Mehrafarin et al., 2010a; Patel et al., 2023b). However, critical perspectives emphasize the need for integrative strategies that combine classical breeding with modern genomic tools such as GWAS, SNP genotyping, and CRISPR-based gene editing to accelerate cultivar development (Bal et al., 2022; Chaudhary et al., 2018a). *In vitro* systems like callus, suspension, and hairy root cultures have shown considerable promise in enhancing the biosynthesis of alkaloids and saponins under controlled elicitor-induced conditions (Ramesh et al., 2010; Mishra et al., 2021), which may be further scaled via bioreactor platforms. Despite these advances, several research gaps persist. Fenugreek still lacks a fully sequenced and annotated reference genome, which limits gene discovery and targeted functional genomics. Moreover, protoplast regeneration protocols remain inefficient, thereby constraining transformation efficiency and hindering gene editing applications (Zhao et al., 2022). Although CRISPR and biolistic tools have revolutionized legume biotechnology, their application in fenugreek remains largely untapped. Likewise, retrotransposon-based iPBS markers, though promising, have not been sufficiently exploited for genetic mapping. Additionally, while many studies focus on agronomic and abiotic stress traits, molecular insights into biotic stress resistance—such as pest and disease tolerance—are notably underrepresented. There is also a need for better integration of genotypic and metabolomic data to develop predictive models that can correlate gene expression with phytochemical accumulation, thereby enabling more precise selection of elite lines. Addressing these gaps through multidisciplinary strategies will be essential for unlocking the full potential of fenugreek as a functional food and medicinal crop.

## 9 Biotechnological advancements in fenugreek

### 9.1 *In vitro* culture

The *in vitro* culture of fenugreek (*Trigonella foenum-graecum* L.) plays a pivotal role in enhancing its bioactive potential and genetic improvement (Khawar et al., 2004b). Fenugreek is a rich source of derivatives of bioactive compounds, including alkaloids, saponins, choline, steroidal sapogenins, trigonelline, trigocoumarin, and trimethyl (Aasim et al., 2014b). While conventional breeding has led to fenugreek varieties with improved physical traits, climate adaptability, and higher yields, the primary focus remains the enhancement of its bioactive constituents (Radwan and Kokate, 1980a). Understanding variability in metabolite production and their therapeutic pathways is crucial for the genetic advancement of this medicinally significant plant (Al-Habori and Raman, 2002a). *In vitro* plant cell and tissue culture techniques, such as elite plant production, callus induction, cell suspension cultures, somatic embryogenesis, and genetic transformation, have been extensively explored for the commercial synthesis of economically important secondary metabolites like diosgenin and trigonelline (Aasim et al., 2014b; Oncina et al., 2000a; Ramesh et al., 2010). Studies indicate that *in vitro* culture offers a more efficient and consistent method for isolating secondary metabolites than whole-plant or field-grown seed extraction. The controlled environment of *in vitro* systems ensures enhanced metabolite stability and yield while minimizing environmental influences (Rezaeian, 2011a). Additionally, the ability to regulate metabolite concentrations is significantly improved through the strategic use of chemical elicitors, enzymes, organic compounds, and tailored growth conditions (Rezaeian, 2011b). Recent advancements have demonstrated the application of diverse plant tissue culture techniques, including organogenesis, callus culture, cell suspension culture, and protoplast culture, to optimize genetic enhancement and phytochemical biosynthesis (Radwan and Kokate, 1980b; Mishra et al., 2021). Such methodologies not only facilitate large-scale production of bioactive compounds but also open new avenues for biotechnological interventions, ensuring sustainable and high-yielding fenugreek cultivars with enhanced medicinal and nutritional properties.

### 9.2 Cell suspension culture *in vitro*

Cell suspension culture has emerged as a pivotal biotechnological approach for the large-scale synthesis of secondary metabolites, offering a controlled environment to investigate the influence of various organic and chemical compounds on cell proliferation and metabolic pathways (Prabakaran and Ravimycin, 2012). This technique is particularly beneficial in medicinal plant research, where optimizing bioactive compound production is essential for pharmaceutical and nutraceutical applications. In fenugreek (*Trigonella foenum-graecum* L.), cell suspension cultures have been instrumental in enhancing the biosynthesis of steroidal sapogenins, alkaloids, and flavonoids, providing a scalable alternative to traditional plant-based extraction methods (Tsiri et al., 2009b).

Early studies by Cerdon et al. (1995) demonstrated that supplementing fenugreek suspension cultures with 125 μM diniconazole for 21 days resulted in a 20% reduction in cell growth compared to control conditions, alongside a significant 50% decrease in total sterol content, highlighting the compound’s inhibitory effect on plant metabolism. In contrast, Khanna et al. (1975b) successfully enhanced sapogenin accumulation by incorporating cholesterol into the culture medium, a strategy that was further supported by Brain and Williams (1983a), who reported a substantial increase in sapogenin yield through cholesterol supplementation in fenugreek cell suspension systems. Similarly, Trisonthi et al. (1980a) established the positive impact of mevalonic acid on steroidal sapogenin biosynthesis, reinforcing the critical role of precursor feeding in secondary metabolite production.

Beyond steroidal compounds, cell suspension cultures have also facilitated the targeted enhancement of other phytochemicals. Tsiri et al. (2009a) demonstrated a strong correlation between copper supplementation and the *de novo* synthesis of medicarpin, a bioactive isoflavonoid pterocarpan with potent antioxidant and antimicrobial properties. Furthermore, Ramesh et al. (2010) observed that the addition of nicotinic acid to fenugreek cell suspension cultures led to a 37% increase in trigonelline content, a key alkaloid with hypoglycemic and neuroprotective effects. These findings align with recent advancements in metabolic engineering and elicitor-based strategies, where exogenous application of precursors, signaling molecules, and stress-inducing agents has been shown to upregulate biosynthetic pathways.

More recent studies have expanded upon these foundational findings. Mishra et al. (2021) explored the application of elicitors such as jasmonic acid and salicylic acid in cell suspension cultures, revealing their capacity to enhance alkaloid and flavonoid accumulation in medicinal plants, including fenugreek. Additionally, highlighted the integration of bioreactor-based suspension culture systems, which offer a promising avenue for large-scale, cost-effective metabolite production. This research underscores the growing potential of *in vitro* cell suspension cultures not only as a tool for understanding plant metabolic pathways but also as an efficient system for sustainable bioactive compound synthesis.

### 9.3 Protoplast culture *in vitro*

Despite the significant progress in plant tissue culture, research on fenugreek (*Trigonella foenum-graecum* L.) protoplast culture remains limited (Shekhawat and Galston, 1983). This technique, primarily employed for shoot regeneration and the *in vitro* isolation of secondary metabolites, offers immense potential for genetic transformation, metabolite enhancement, and somatic hybridization. However, establishing an efficient and reproducible protocol for protoplast culture in fenugreek has proven challenging due to its recalcitrant nature and the difficulties associated with cell wall regeneration and sustained division (Zhao et al., 2022).

One of the earliest breakthroughs in fenugreek protoplast culture was reported by Shekhawat and Galston (1983), who successfully induced green calli and leafy shoots from mesophyll protoplasts derived from leaf explants. These protoplasts were cultivated on a medium supplemented with 0.1 mg/L 6-Benzylaminopurine (BAP) and Zeatin, demonstrating the role of cytokinins in shoot organogenesis. While Christen (2002a) achieved successful protoplast culture using root apices explants, their attempts to induce shoot regeneration were unsuccessful, highlighting the species-specific and explant-dependent nature of protoplast totipotency. In contrast, Petropoulos (2002a) and Mehrafarin et al. (2010b) documented successful shoot induction from protoplasts derived from root apices, suggesting that optimized hormonal balance and culture conditions are crucial for overcoming regeneration barriers.

Beyond regeneration, fenugreek protoplast culture has shown promise in metabolite biosynthesis (Christen, 2002b). Callus tissues generated from protoplast cultures exhibited significantly enhanced secondary metabolite accumulation, with trigonelline concentrations found to be three to four times higher than in seeds and nearly twelve to thirteen times higher than in roots and shoots (Trisonthi et al., 1980b). This aligns with broader trends in plant biotechnology, where dedifferentiated cells, such as callus and suspension cultures, often exhibit higher metabolic plasticity, facilitating enhanced production of bioactive compounds (Gantait et al., 2021b).

Recent advancements in plant protoplast culture techniques, including microfluidic culture systems and nanotechnology-based approaches, have opened new avenues for improving protoplast viability and regeneration efficiency (Zhao et al., 2022). Additionally, CRISPR-Cas9-mediated genome editing in protoplasts has been successfully applied to enhance stress tolerance and secondary metabolite biosynthesis in medicinal plants, a strategy that could potentially be extended to fenugreek (Jiang et al., 2020). Future research should focus on refining protoplast isolation protocols, optimizing osmotic stabilizers, and exploring bioreactor-based culture systems to maximize regeneration success and metabolite production.

### 9.4 Callus culture *in vitro*

Callus culture plays a pivotal role in fenugreek (*Trigonella foenum-graecum* L.) research, serving as a foundational technique for various *in vitro* processes, including protoplast culture, cell suspension culture, somatic embryogenesis, proliferation, and the biosynthesis of secondary metabolites (Ahmed et al., 2000). Despite its potential for plant regeneration, studies on fenugreek callus culture have primarily focused on enhancing metabolite production rather than shoot proliferation, underscoring its significance in pharmaceutical and industrial applications (Ahmed et al., 2000).

Comparative studies have demonstrated that callus induction, as opposed to direct seed germination, is a more cost-effective and efficient approach for secondary metabolite biosynthesis (Azam and Biswas, 1989). This efficiency stems from the ability to manipulate explant sources, optimize plant growth regulator (PGR) combinations, and refine culture conditions to maximize yield (Singh et al., 2022; Gantait et al., 2021a). One of the earliest breakthroughs in callus-mediated metabolite enhancement was reported by Joshi and Handler (1960), who highlighted the role of nicotinic acid and S-adenosylmethionine in trigonelline biosynthesis. Their findings revealed that supplementing the culture medium with ATP and MgCl_2_ resulted in a three- to four-fold increase in trigonelline concentration compared to seeds. Similarly, Khanna and Jain (1973) demonstrated that specific growth conditions significantly boosted steroidal content in callus cultures, leading to higher accumulations of diosgenin, gitogenin, and tigogenin—key precursors in steroidal drug synthesis.

Further supporting the superior metabolic activity of callus cultures, Radwan and Kokate (1980a) reported trigonelline yields surpassing those found in seed, root, and shoot cultures. These findings were later corroborated by Ahmed et al. (2000) and Oncina et al. (2000b), who demonstrated that fenugreek callus consistently produced higher concentrations of trigonelline and diosgenin compared to *in vivo* cultures. Additionally, Rezaeian (2011a) established that increasing 2,4-D concentrations significantly enhanced callus induction, with shoot apical meristem explants yielding the most robust calli.

Explant selection and culture conditions have emerged as key determinants of metabolite synthesis. Ciura et al. (2015a) identified leaves as the most metabolically active plant organ and emphasized the influence of harvesting time and media composition on diosgenin concentration. Meanwhile, Alalwani and Alrubaie (2016) explored the synergistic effects of polyethylene glycol (PEG) and magnetized water in trigonelline biosynthesis, identifying optimal conditions for maximizing production.

Beyond metabolite synthesis, extensive research has aimed at optimizing callus induction for plant regeneration. Shekhawat and Galston (1983); Azam and Biswas (1989), and El-Bahr (1989) were among the first to establish specific culture media compositions that facilitated effective callus initiation and growth. More recently, Abd Elaleem et al. (2014) successfully induced callus from cotyledon and hypocotyl explants, while Seyedardalan and Mahmood (2013) reported direct somatic embryogenesis from hypocotyls—an achievement that holds promise for genetic transformation studies. Despite these advancements, achieving efficient shoot induction from callus remains a persistent challenge. Aasim et al. (2010a) and El-Nour et al. (2015) highlighted the complexities of shoot regeneration, noting that while callus induction was frequently successful, translating this into complete plant development posed difficulties.

As fenugreek continues to gain recognition for its medicinal and nutritional benefits, research efforts aimed at optimizing callus culture parameters remain critical (Pant et al., 2013). Integrating modern biotechnological tools, such as metabolic engineering and bioreactor-based callus cultivation, could further enhance metabolite yields while refining protocols for shoot regeneration (Patel et al., 2023b; Zhao et al., 2022). These advancements will not only strengthen fenugreek’s role in the pharmaceutical industry but also contribute to its sustainable production through *in vitro* culture systems.

### 9.5 *In vitro* regeneration and organogenesis

For researchers striving to develop a dependable and repeatable technique, fenugreek *in vitro* organogenesis presents a formidable challenge (Kavcı et al., 2017). Despite extensive efforts, the natural resistance of fenugreek to *in vitro* regeneration continues to hinder scientific progress (Elaleem et al., 2014; Prabakaran and Ravimycin, 2012). Numerous studies have explored direct and indirect organogenesis, as well as somatic embryogenesis, yet significant hurdles remain—particularly in root development, proliferation, and plantlet acclimatization (Seyedardalan et al., 2013). Consequently, researchers have increasingly turned to alternative approaches, such as callus induction, cell suspension cultures, and protoplast cultures, to enhance secondary metabolite production and genetic modification potential (Al-Alwani and Alrubaie, 2016; Taşbaşi et al., 2017).

Khawar et al. (2004a) achieved shoot induction from apical meristem explants but faced limitations in rooting plantlets, highlighting a fundamental issue in fenugreek micropropagation. Similarly, Aasim et al. (2010b) experimented with various explants and plant growth regulators (PGRs) for shoot regeneration, demonstrating promising results from cotyledonary node explants. However, the inability to establish rooted plantlets remained a major limitation. Afsharie et al. (2011) further investigated basal medium salts and explant types for *in vitro* regeneration, identifying optimal conditions for somatic embryogenesis and shoot formation. However, challenges persisted in plantlet establishment.

Prabakaran and Ravimycin (2012) reported multiple shoot induction from shoot tip explants, but their study lacked information on root development and adaptation to *ex vitro* conditions. Vaezi et al. (2015) underscored the critical role of explant age and sucrose concentration in regeneration efficiency.

Recent advancements have provided deeper insights into fenugreek’s tissue culture responses. Taşbaşi et al. (2017) and Kavci et al. (2017) examined the effects of sucrose concentration, explant age, and explant type on regeneration, reporting successful callus induction and somatic embryogenesis. However, Taşbaşi et al. (2017) noted failures in shoot induction from leaf explants, while Kavci et al. (2017) achieved shoot bud development under optimized conditions. Complementary studies by highlighted the influence of auxins and cytokinins in balancing callus formation and shoot organogenesis. Moreover, Patel et al (2023b) explored bioreactor-based strategies, which offer a scalable alternative for metabolite production and genetic transformation research (Petropoulos, 2002b).

Fenugreek has greatly benefited from advancements in plant cell and tissue culture techniques, particularly for secondary metabolite production and genetic modification applications (Vaezi et al., 2015). However, the persistent difficulties in achieving fully functional and adaptable *in vitro* plantlets reflect the intrinsic recalcitrance of fenugreek (Taşbaşi et al., 2017). Future research should focus on refining rooting protocols, improving explant responses, and integrating molecular tools to enhance regeneration efficiency (Vaezi et al., 2015). Addressing these bottlenecks will unlock new possibilities for incorporating genes of interest and advancing biotechnological applications in fenugreek research.

### 9.6 Genetic transformation

The application of genetic transformation in medicinal plants has gained significant traction in recent years, driven by the quest to enhance the biosynthesis of bioactive compounds with both therapeutic and commercial value (Seyedardalan et al., 2013). However, fenugreek (*Trigonella foenum-graecum* L.) remains relatively understudied in this domain, with limited studies utilizing *Agrobacterium tumefaciens* or *Agrobacterium rhizogenes* for genetic modifications. Among the few successful endeavors, *A. rhizogenes* has been employed to induce hairy root cultures, primarily to augment the production of key secondary metabolites such as diosgenin and trigonelline. Despite the limited scope of research, these studies have demonstrated the potential of various *A. rhizogenes* strains in facilitating hairy root development and enhancing metabolite content (Merkli et al., 1997; Raheleh et al., 2011).

Merkli et al. (1997) pioneered the establishment of hairy root cultures in fenugreek by infecting sterile 2-week-old plantlet stems with *A. rhizogenes* strain A4. Their study assessed hairy root proliferation and diosgenin accumulation across different culture media (WP, MS, and B5), revealing differential metabolite accumulation influenced by nutrient composition. Similarly, Raheleh et al. (2011) investigated the impact of multiple *A. rhizogenes* strains and infection strategies, achieving a remarkable 100% hairy root induction rate. Their findings highlighted significant trigonelline accumulation, with measured levels reaching 14.89 mg g^-^¹ DW in the Borazjan variety after 28 days and 14.03 mg g^-^¹ DW in the Zanjan variety within just 7 days.

Beyond secondary metabolite production, *A. rhizogenes* has also been employed as a tool for gene expression studies in fenugreek. Shahabzadeh et al. (2013) utilized *A. rhizogenes* strain K599 carrying a green fluorescent protein (GFP) gene to assess transformation efficiency. Their findings underscored the critical role of plant genotype, bacterial strain type, explant selection, and inoculation parameters in achieving successful genetic modifications. In contrast, the application of *A. tumefaciens* in fenugreek transformation remains exceptionally rare. One of the few reported cases comes from Khawar et al. (2004a), who used *A. tumefaciens* strain A281—carrying the β-glucuronidase (GUS) gene—to transform cotyledon, root, and hypocotyl explants from one-week-old seedlings. PCR amplification confirmed the presence of the *uidA* gene, while histochemical assays validated GUS gene expression in tumor-induced tissues.

Despite these advancements, the genetic transformation of fenugreek remains in its infancy. Notably, there is a conspicuous absence of research incorporating agronomically significant transgenes, such as those conferring herbicide tolerance or insect resistance. Additionally, alternative transformation methods—such as biolistic bombardment and protoplast-based approaches—have yet to be explored, likely due to the absence of a standardized tissue culture protocol, difficulties in root development, and uncertainties surrounding transformation efficiency (Jha et al., 2020; Singh et al., 2021). Addressing these challenges will be pivotal in unlocking fenugreek’s full biotechnological potential, paving the way for enhanced genetic improvements and commercial applications.

## 10 Omics in fenugreek

**Figure 1:**
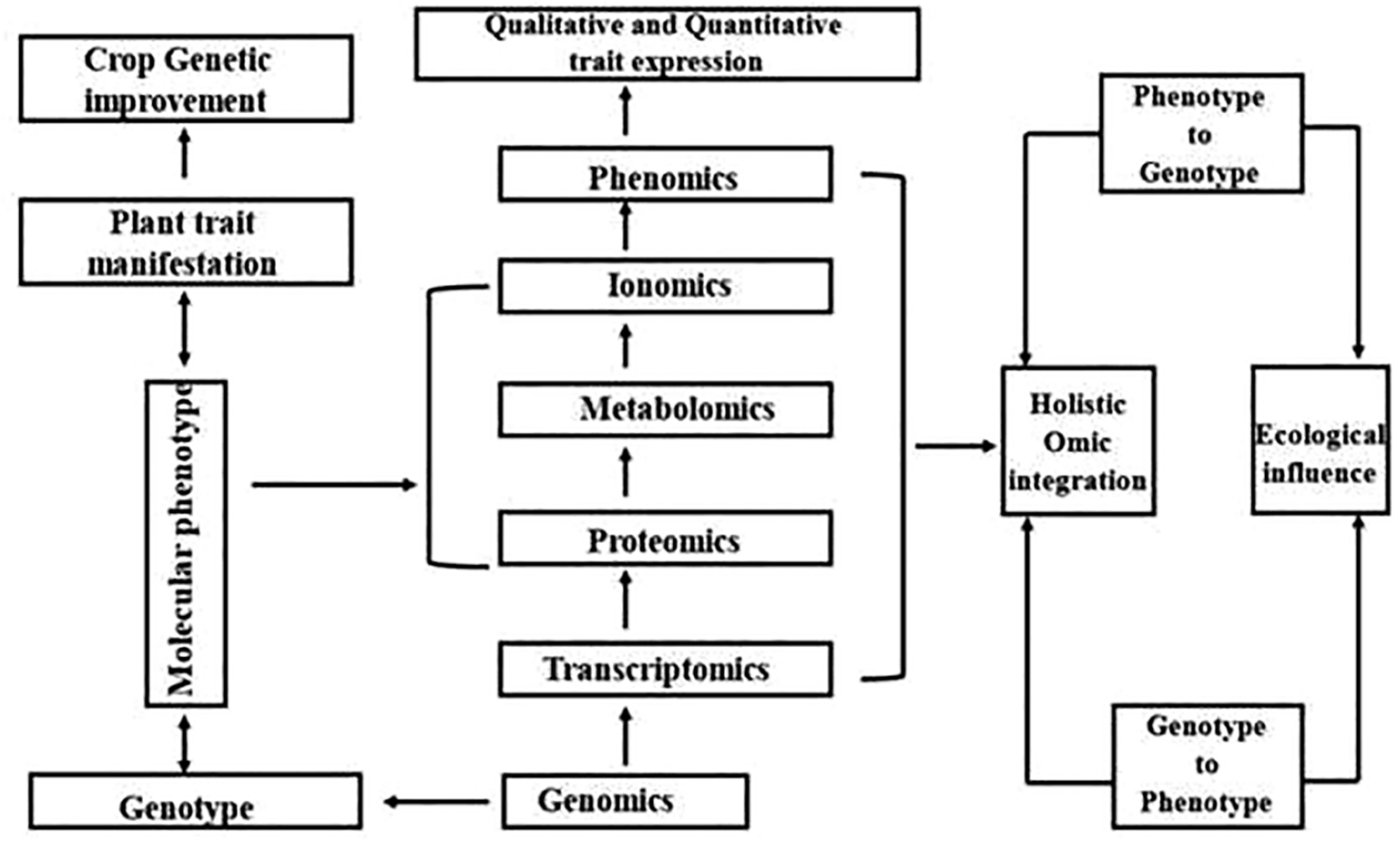
A glimpse into omics.

The rapid evolution of biotechnology has given rise to the Omics sciences, a collective term that encompasses genomics, transcriptomics, proteomics, and metabolomics (**Figure 1**). These interrelated fields provide a comprehensive, multi-layered understanding of biological systems—from individual cells to entire organisms. When integrated, they form the foundation of systems biology, an approach that emphasizes the holistic study of complex biological networks (Horgan and Kenny, 2011). This paradigm shift moves beyond reductionist methodologies, allowing for a more intricate exploration of life at the molecular level.

Unlike traditional hypothesis-driven research, Omics methodologies generate vast datasets that enable data-driven hypothesis formulation (Kell and Oliver, 2004). These insights serve as the groundwork for further experimental validation, offering critical perspectives on physiological mechanisms, metabolic pathways, and molecular markers associated with desired traits. Their applications extend across diverse scientific fields, from medical diagnostics and biomarker discovery to plant biotechnology and precision breeding. By deciphering host-pathogen interactions and identifying genes responsible for specific phenotypic traits, Omics technologies accelerate the development of improved plant varieties, surpassing the limitations of conventional breeding techniques. However, despite these advantages, eukaryotic organisms present inherent challenges—such as complex gene regulatory networks and multifaceted metabolic pathways—that complicate data interpretation and functional annotation (Mochida and Shinozaki, 2011).

Technological advancements in Omics have revolutionized functional genomics, granting researchers an unprecedented edge in understanding biological processes. Genomics focuses on whole-genome analysis, decoding genetic information to unravel the blueprint of life. Transcriptomics investigates gene expression patterns, identifying genes linked to specific biological functions. Proteomics delves into protein interactions and their roles in cellular processes, while metabolomics examines biochemical signatures and metabolic alterations in response to internal or external stimuli (Agrawal et al., 2015). These integrated approaches provide a deeper understanding of genotype-phenotype relationships, enhancing agricultural productivity, stress resilience, and plant quality. From genetic modifications to fundamental physiological discoveries, Omics-driven research continues to reshape the landscape of modern biotechnology (Gupta et al., 2019c).

**Figure 2:**
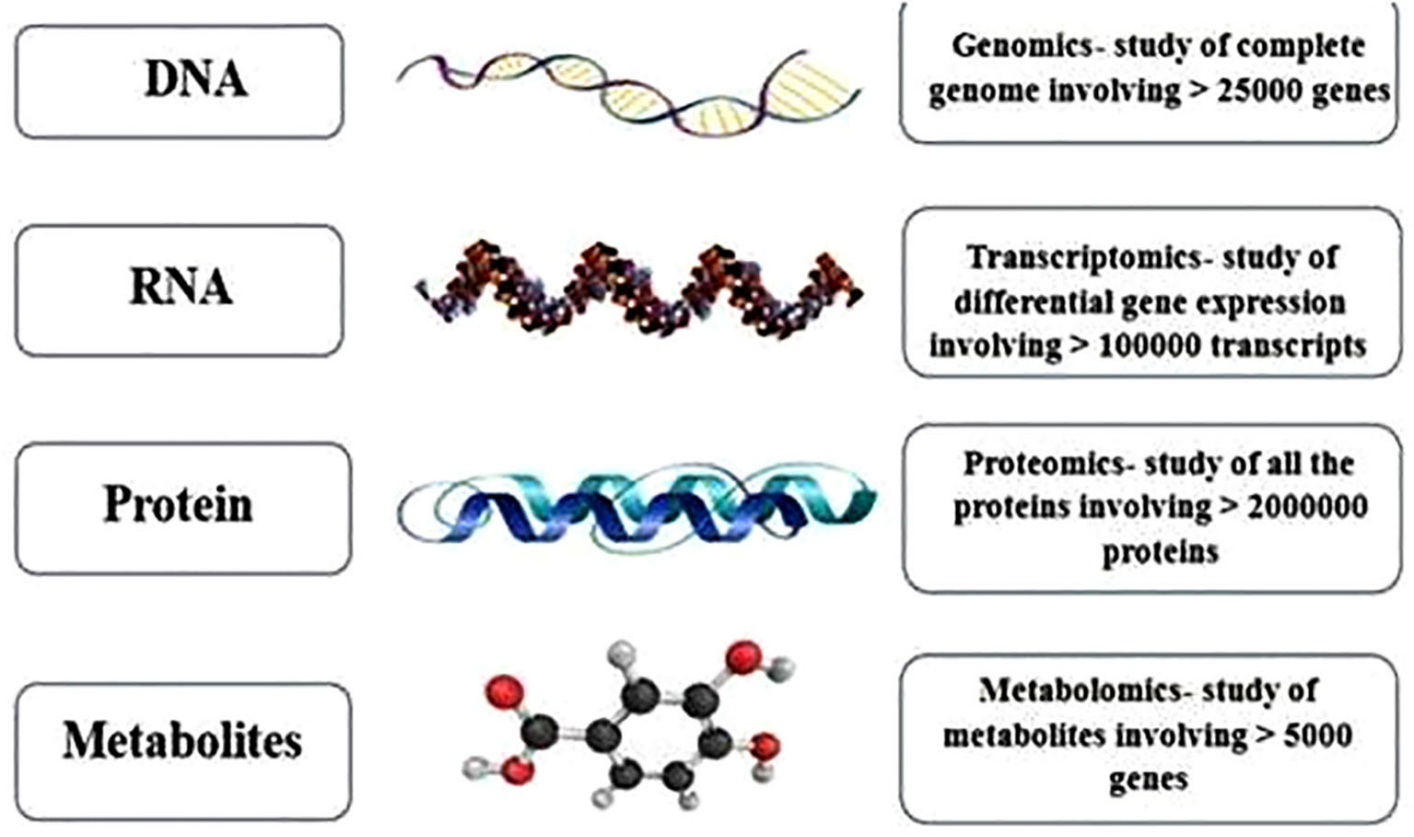
The figures provided represent approximate quantities at each level: DNA is transcribed into RNA, RNA is translated into protein, and proteins are converted into various metabolites through multiple biological processes.

Tracing the journey from DNA to metabolites (**Figure 2**), Omics methodologies have been extensively applied to various crops. However, their potential in *Trigonella foenum-graecum* (fenugreek)—a medicinally and nutritionally valuable legume—remains relatively underexplored. So far, fewer than 20 peer-reviewed Omics-based studies have specifically focused on fenugreek, including transcriptomic investigations into diosgenin biosynthesis pathways, proteomic analyses of stress-responsive proteins, and metabolomic profiling of bioactive compounds (Chaudhary et al., 2018b; Gupta et al., 2019a; Ciura et al., 2015b). This limited body of work underscores both the promise and the pressing need for deeper Omics-based exploration in fenugreek to complement traditional breeding and biochemical studies. Nevertheless, emerging research has yielded promising results, paving the way for novel insights into its genetic architecture, metabolic pathways, and bioactive compound synthesis (Chaudhary et al., 2018c). The following sections provide an in-depth exploration of fenugreek-based Omics studies, highlighting key discoveries and future research prospects.

### 10.1 Genomics

**Figure 3:**
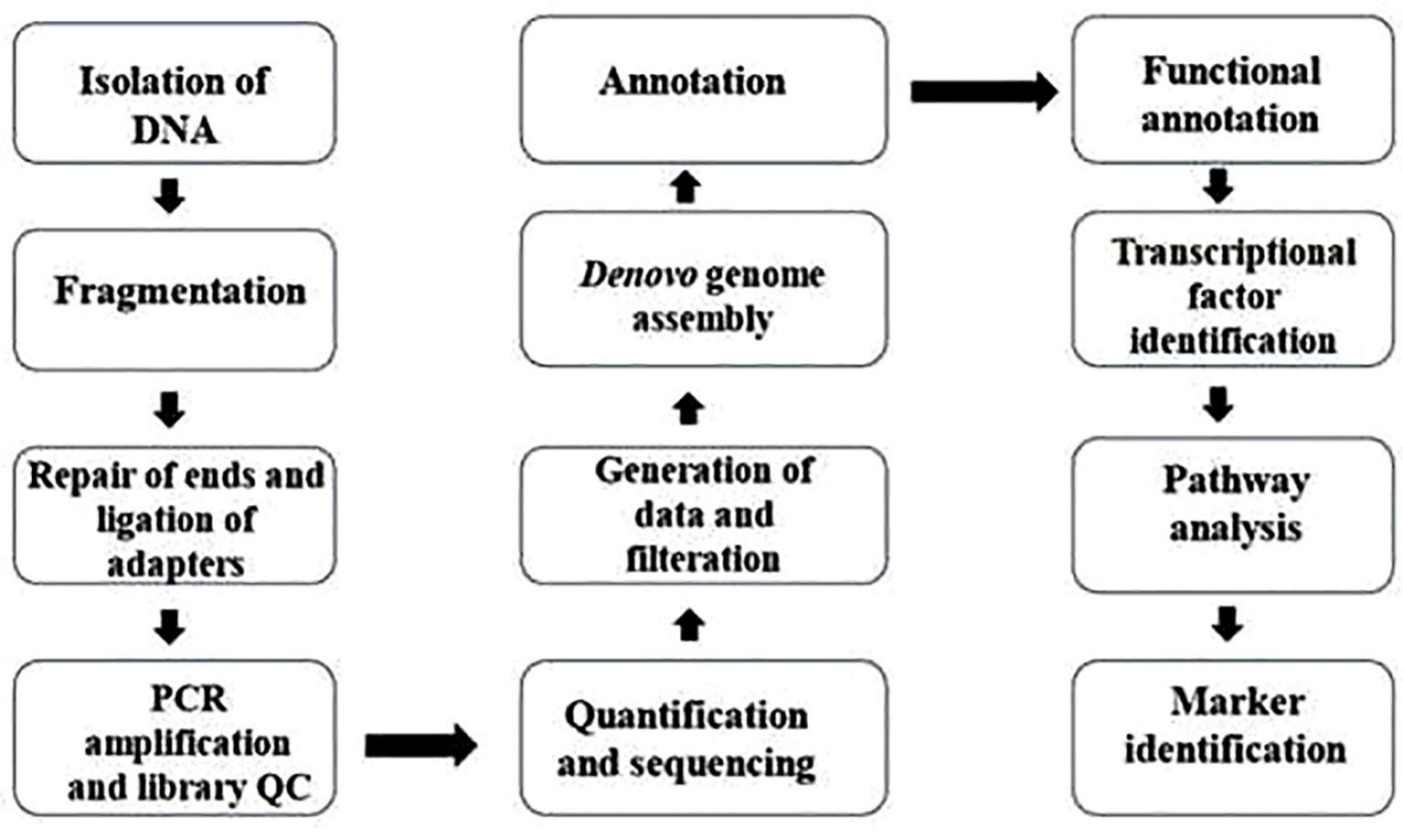
The flowchart depicts the process of whole genome analysis. The steps outlined in this flowchart are typical procedures utilized in preparing shotgun/paired-end genome sequencing libraries and conducting their bioinformatics analysis.

Genomics, the systematic study of an organism’s entire DNA sequence, provides critical insights into its genetic architecture, evolutionary relationships, and functional traits (Shi et al., 2011). A fundamental prerequisite for any genomic exploration is the assessment of genome size, which represents the total DNA content within a haploid set of chromosomes. Flow cytometry remains a widely adopted technique for genome size estimation, leveraging relative fluorescence intensity to quantify DNA content (**Figure 3**). This approach relies on a reference standard with a known genome size to compare and determine the genome size against which the genome size of unknown samples can be compared and determined (Doležel and Bartoš, 2005).

Fenugreek (*Trigonella foenum-graecum*), a medicinally valuable legume, has been estimated to possess a genome size of approximately 0.7 C-value—making it 1.5 times larger than those of model legumes such as *Medicago truncatula* (470 Mbp) and *Lotus japonicus* (685 Mbp) (Vaidya et al., 2013; Young et al., 2003). Despite its therapeutic significance, the complete genome of fenugreek remains unsequenced. The limited karyotypic data available suggests that species within the *Trigonella* genus exhibit diverse somatic chromosome counts (2n = 14, 16, 30, 46), along with the presence of B chromosomes, which could play a role in genetic variation and adaptability (Martin et al., 2011a).

Molecular characterization studies of fenugreek have leveraged a range of DNA-based markers, including Random Amplified Polymorphic DNA (RAPD), Simple Sequence Repeats (SSR), Amplified Fragment Length Polymorphism (AFLP), and Restriction Fragment Length Polymorphism (RFLP) (Shi et al., 2011). These molecular tools have facilitated the genetic differentiation among fenugreek cultivars. Notably, Kasuri methi (*Trigonella corniculata*), a fenugreek variant prized for its aromatic qualities in South Asian cuisine, has been found to exhibit distinct genetic and phenotypic traits. It differs significantly from other fenugreek cultivars in terms of leaf size, plant height, and metabolic profile (Spandan and Pooja, 2019). Comparative gene expression analyses further underscore its genetic divergence from standard fenugreek varieties.

Beyond classification, genomic research holds immense potential for identifying key genes and molecular markers associated with agronomic traits, yield optimization, and resilience against biotic and abiotic stresses. Deciphering the fenugreek genome could pave the way for targeted breeding strategies, improved crop performance, and enhanced medicinal applications (Varshney and Dubey, 2009b). Just as genomic sequencing has revolutionized research on other medicinal plants, unraveling the genetic blueprint of fenugreek will be instrumental in harnessing its full potential for agricultural and pharmaceutical advancements. Recent efforts by Yadav et al. (2024) have further contributed to the understanding of fenugreek genomics by providing genome-wide molecular insights, including marker-trait associations and candidate gene predictions based on in silico tools. Although genome-wide resources for fenugreek remain sparse, their study offers foundational information that can serve as a valuable reference point for future sequencing, gene discovery, and genome annotation projects. Incorporating this kind of species-specific genomic analysis is critical to avoid the over-generalization of genetic data across legume species, which can lead to misleading conclusions. Thus, inclusion of such focused work strengthens the specificity of genomics-related discussions in fenugreek.

### 10.2 Transcriptomics

The transcriptome represents the complete set of RNA molecules in a cell at a specific time, serving as a dynamic reflection of gene activity. Studying transcriptomics allows researchers to decipher the transcriptional architecture of genes, including their 5′ and 3′ terminal sites, splicing variations, and post-transcriptional modifications (Pandit et al., 2018). Early transcriptomic studies relied on conventional techniques such as northern blotting, nylon membrane arrays, and reverse transcriptase quantitative PCR (RT-qPCR) (Becker-Andre and Hahlbrock, 1989; Pandit et al., 2018). However, the advent of high-throughput sequencing has revolutionized transcriptomics, enabling unprecedented insights into gene expression patterns and metabolic pathways.

Advancements in next-generation sequencing (NGS) technologies have significantly accelerated large-scale plant genome and transcriptome research. A critical aspect of transcriptome analysis is capturing the expression profiles of genes associated with key metabolites, leading to the discovery of novel biosynthetic pathways. The biosynthesis of diosgenin in *Trigonella foenum-graecum* follows a highly regulated and compartmentalized metabolic route, primarily derived from the mevalonate (MVA) pathway. This pathway initiates with acetyl-CoA conversion to isopentenyl pyrophosphate (IPP) via a series of reactions catalyzed by enzymes like 3-hydroxy-3-methylglutaryl-CoA reductase (HMGR)—a key regulatory gene in the MVA pathway. IPP units condense to form squalene, mediated by squalene synthase (SQS), which is subsequently converted into cholesterol-like precursors (Khanna et al., 1975a). These sterol intermediates undergo multiple oxidation and hydroxylation steps, catalyzed by various cytochrome P450 monooxygenases (CYP450s), to ultimately yield diosgenin, a major steroidal sapogenin. Integration of transcriptomic data has revealed differential expression of these key genes (e.g., HMGR, SQS, CYP90B, CYP72A) under specific growth stages and elicitor treatments, offering insight into the molecular regulation of diosgenin biosynthesis (Mehrafarin et al., 2010c; Patel et al., 2023b; Chaudhary et al., 2018a). These findings provide a mechanistic framework for metabolic engineering and targeted breeding strategies aimed at enhancing diosgenin yield in fenugreek. The ability of fenugreek (*Trigonella foenum-graecum*) to adapt to abiotic stress conditions such as drought and salinity is mediated by complex molecular networks involving stress perception, signal transduction, and transcriptional reprogramming (Pant et al., 2013). Upon exposure to environmental stress, specific membrane-bound receptors and sensors initiate signaling cascades that activate downstream transcription factors (TFs), notably members of the MYB, WRKY, and NAC families. These TFs regulate stress-responsive genes involved in osmolyte biosynthesis (e.g., proline, trehalose), antioxidant enzyme production (e.g., SOD, CAT, APX), and modulation of root architecture for improved water uptake. Transcriptome profiling of fenugreek under stress conditions has revealed differential expression of genes related to reactive oxygen species (ROS) detoxification, hormone signaling (e.g., ABA and JA), and cell wall remodeling, highlighting their roles in conferring phenotypic resilience (Gupta et al., 2019a; Chaudhary et al., 2018a; Patel et al., 2023b). Integration of omics data (transcriptomics and proteomics) with physiological observations supports the existence of tightly coordinated regulatory networks that govern fenugreek’s adaptation mechanisms, offering valuable targets for functional genomics and stress-resilient breeding programs. Among these innovations, RNA sequencing (RNA-seq) has emerged as a powerful, cost-effective alternative to microarrays, especially in the absence of complete genome sequencing data. By directly sequencing complementary DNAs (cDNAs), RNA-seq generates high-resolution short reads that can be assembled into comprehensive transcriptional profiles. This approach not only provides qualitative and quantitative insights into transcriptomes but also facilitates the identification of novel exons, alternative splicing events, and regulatory elements (Shi et al., 2011; Chikara et al., 2014).

Fenugreek (*Trigonella foenum-graecum*) has been recognized for over five centuries for its dual applications in culinary and medicinal fields (Chaudhary et al., 2018a). Historically, traditional Indian and Chinese medicine has utilized fenugreek for treating ailments such as paralysis, gout, chronic cough, diabetes, sinus infections, inflammation, and even hair loss (Chaudhary et al., 2015; Mullaicharam et al., 2013a). Recent pharmacological studies further highlight its broad spectrum of biological activities, including antibacterial, antiparasitic, anticancer, antifertility, anti-aging, galactagogue, and hypocholesterolemic properties (Chaudhary et al., 2015; Al-Asadi, 2014). These diverse medicinal properties are largely attributed to fenugreek’s complex biochemical composition, particularly its secondary metabolites. Among them, saponins such as gitogenin, diosgenin, yamogenin, tigogenin, and neotigogenin play key therapeutic roles.

Diosgenin, a steroidal saponin, is of particular significance due to its role as a precursor in the synthesis of over 200 steroidal drugs, including glucocorticoids, testosterone, progesterone, and oral contraceptives (Zhou et al., 2019). Despite its pharmaceutical importance, the complete biosynthetic pathway of diosgenin remained poorly understood until transcriptome sequencing efforts shed light on the genetic framework underlying its production. Early research on steroid biosynthesis was largely limited to understanding cholesterol and sitosterol synthesis from cycloartenol-derived precursors. The advent of RNA sequencing marked a turning point, enabling comprehensive investigations into fenugreek’s biosynthetic machinery (Vaidya et al., 2013; Mehrafarin et al., 2010d).

Mehrafarin et al. (2010a) were among the first to propose a mechanistic pathway for cholesterol synthesis from acetyl-CoA, identifying the involvement of 11 key enzymes. Building upon this foundation, Vaidya et al. (2013) leveraged RNA sequencing to delineate the complete steroid biosynthesis pathway in fenugreek. Their study revealed that diosgenin synthesis involves three interconnected biochemical routes: the glycolytic pathway, the mevalonate pathway, and the steroid biosynthesis pathway. They further identified two potential routes for diosgenin formation from squalene 2,3-oxide—one through lanosterol leading to cholesterol, and another via cycloartenol leading to sitosterol. This groundbreaking study, conducted using the SOLiD 4 Genome Analyzer, generated 42 million high-quality reads and led to the functional annotation of 18,333 transcripts, marking the first in-depth characterization of the diosgenin biosynthetic pathway in fenugreek.

The integration of transcriptomic data with metabolic pathway analysis holds great promise for enhancing our understanding of fenugreek’s pharmaceutical potential. Future studies leveraging advanced omics technologies will likely unveil additional regulatory mechanisms governing secondary metabolite biosynthesis, paving the way for genetic improvements and biotechnological advancements in medicinal plant research.

### 10.3 Proteomics in fenugreek

Proteomics is the comprehensive analysis of the entire protein repertoire within a defined biological context, such as a tissue, cell, or subcellular compartment. As pivotal players in major metabolic and signaling pathways, proteins are fundamental to understanding the molecular mechanisms governing plant growth, development, and environmental interactions (Jacobs et al., 2000). Proteomics research enables the investigation of protein structure, function, and dynamics, offering crucial insights into cellular and biochemical processes (Jacobs et al., 2000; Varshney and Dubey, 2009a; Aebersold and Mann, 2016).

A core aspect of proteomics involves protein separation, detection, and functional characterization. One of the most established methods for protein separation is two-dimensional gel electrophoresis (2-DGE). This technique employs isoelectric focusing (IEF) to separate proteins based on their isoelectric points, followed by sodium dodecyl sulfate polyacrylamide gel electrophoresis (SDS-PAGE), which further resolves proteins according to molecular weight. Visualization of separated proteins is achieved through staining techniques, such as Coomassie Brilliant Blue, silver nitrate, or fluorescent dyes (Görg et al., 2004). Despite its widespread application, 2-DGE has inherent limitations, including limited effectiveness in resolving hydrophobic and low-abundance proteins. To overcome these challenges, gel-free approaches, such as liquid chromatography coupled with tandem mass spectrometry (LC-MS/MS), have revolutionized proteomics by offering high-throughput and sensitive protein identification (Zhang et al., 2013).

Recent technological advancements have expanded the scope of proteomics beyond basic protein identification (Sethi and Brietzke, 2016b). Modern proteomics now integrates quantitative techniques such as label-free quantification and stable isotope labeling to measure protein abundance dynamically. Additionally, high-throughput strategies facilitate the exploration of protein-protein interactions, subcellular localization, and post-translational modifications (PTMs), which significantly influence protein function and cellular responses (Aebersold and Mann, 2016; Mann and Jensen, 2003). These advancements allow for a more holistic understanding of complex biological systems, bridging gaps between genomics, transcriptomics, and metabolomics.

Although transcriptomics and genomics provide valuable insights into gene expression, they do not always correlate directly with protein abundance. Alternative splicing and mRNA processing events generate multiple protein isoforms from a single transcript, thereby increasing proteomic complexity (Ideker et al., 2001; Wang et al., 2008). Protein function is also modulated by spatial distribution within the cell, interaction with biomolecules, and environmental stimuli. Given the dynamic nature of proteomes, high-resolution proteomics methods are indispensable for capturing the full range of cellular protein diversity.

Proteomics research has made significant strides in model organisms such as Escherichia coli, yeast, and mammals, while plant proteomics has historically lagged. However, the availability of complete genome sequences for model plants, including Arabidopsis thaliana, rice, and poplar, alongside extensive expressed sequence tag (EST) libraries and gene indices for over 31 plant species, is accelerating progress in plant proteomics (Jorrín-Novo et al., 2009; van Wijk, 2001). These resources enable comprehensive proteome mapping, shedding light on plant-specific physiological processes and stress responses.

Proteomics is a rapidly evolving field that offers profound insights into cellular and molecular biology (Jacobs et al., 2000). With the integration of advanced analytical techniques, computational tools, and large-scale proteomic datasets, researchers are now poised to unravel the intricate regulatory networks underlying plant growth, adaptation, and resilience. The synergy between proteomics, genomics, and transcriptomics paves the facilitates groundbreaking discoveries in plant biology, ultimately contributing to agricultural innovations and sustainable crop improvement.

### 10.4 Metabolomics

**Figure 4:**
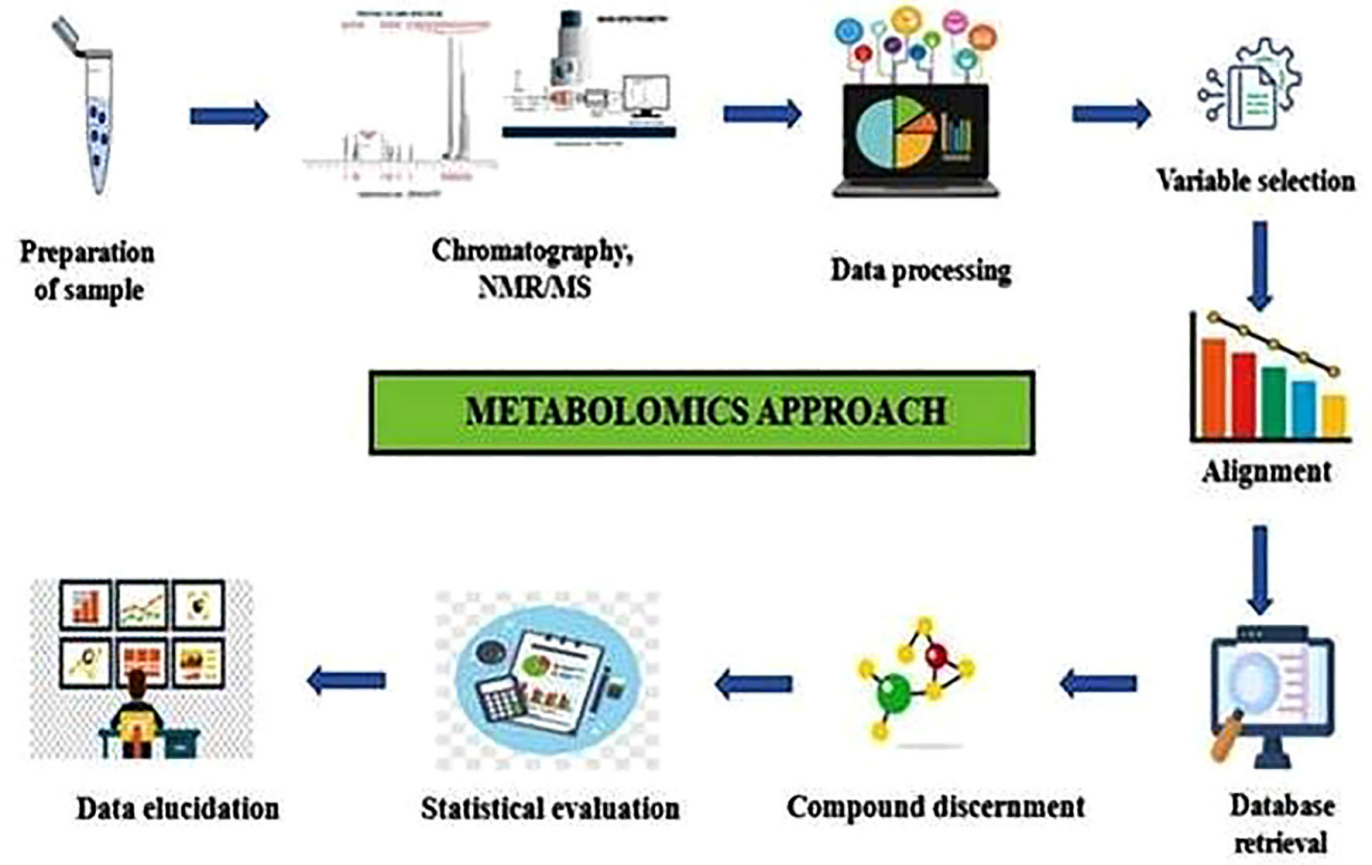
Diagram illustrating the steps of metabolomics studies.

Metabolites serve as the ultimate biochemical signatures of cellular processes, providing a real-time snapshot of a cell’s physiological state (Sethi and Brietzke, 2016a). These small molecules arise from the intricate interplay between an organism’s genome and its environment, not only acting as the end products of gene expression but also playing a crucial role in regulatory networks (Fiehn, 2002). By integrating metabolomics with transcriptomics and proteomics, researchers gain a more holistic understanding of biological systems (**Figure 4**). Unlike genetic or proteomic changes that may take hours or days, metabolic fluctuations can occur within seconds, offering a dynamic perspective on cellular functions (Goodacre et al., 2004; Tan et al., 2016). The ability to study these rapid changes has led to significant advancements in understanding biochemical pathways, metabolic adaptations, and gene function in various organisms.

To analyze and quantify metabolites, researchers employ high-resolution analytical techniques such as nuclear magnetic resonance (NMR) spectroscopy and mass spectrometry (MS) (Tan et al., 2016). MS-based methods, coupled with chromatographic separation techniques like high-performance liquid chromatography (HPLC), enable the identification of complex metabolite profiles with high sensitivity and specificity. The synergy between these techniques allows researchers to explore primary and secondary metabolites across different plant species, including medicinal plants like fenugreek (*Trigonella foenum-graecum*).

Despite the therapeutic importance of fenugreek, metabolomics studies on this plant remain limited, with most research primarily focusing on saponins rather than the complete metabolome (Zhou et al., 2012). The majority of studies utilize fenugreek seeds, although other plant parts—such as leaves, stems, and roots—have also been investigated to a lesser extent. The extraction process generally involves drying and pulverizing the plant material into a fine powder, followed by homogenization with methanol or ethanol under acidic conditions. Hydrolysis at elevated temperatures (>120°C) facilitates metabolite release, and successive extractions using solvents like petroleum ether or n-hexane ensure optimal recovery of bioactive compounds. Analytical techniques such as gas chromatography-mass spectrometry (GC-MS), ultra-performance liquid chromatography coupled with collision-induced dissociation tandem mass spectrometry (UPLC-CID-MS/MS), and HPLC are then employed to profile the extracted metabolites.

Metabolomics-based mass spectrometry studies have elucidated the compositional differences in *Trigonella* species, identifying 93 chromatographic peaks corresponding to diverse phytochemical classes (Tan et al., 2016). These include phenolic acids, fatty acids, nitrogenous dipeptides, steroid/triterpene saponins, and flavonoids. Among these, the presence and abundance of C-flavonoids and saponins appear to be the key differentiating factors influencing the biochemical properties of fenugreek seeds. Additionally, fenugreek contains several free amino acids, such as 4-hydroxy leucine, and volatile compounds including heptanoic acid, n-hexanol, dihydroactinidiolide, dihydrobenzofuran, tetradecane, α-muurolene, β-elemene, and pentadecane. These volatile constituents contribute significantly to the characteristic aroma and bitter taste of fenugreek, while its phytochemicals play a central role in its medicinal attributes.

Despite the strides made in fenugreek research, a comprehensive metabolome profiling remains challenging due to the plant’s chemically diverse metabolite composition, encompassing lipids, organic acids, carbohydrates, amino acids, nucleotides, and steroids. Advanced analytical approaches, particularly MS and NMR spectroscopy, have become indispensable for in-depth metabolomics research. Fourier-transform infrared (FTIR) spectroscopy has also been instrumental in studying biochemical interactions in plant-microbe systems. Additionally, high-performance liquid chromatography-ultraviolet (HPLC-UV) technology, when integrated with robust statistical analyses, has facilitated the profiling of phenolic compounds involved in lignin biosynthesis. MS-based methods further enhance metabolite identification and quantification, boasting remarkable sensitivity, resolution, and dynamic range, capable of detecting molecules at femtomolar to attomolar concentrations. These advancements pave the way for deeper insights into metabolic networks and their functional implications in plant biology and medicinal applications.

As research progresses, integrating metabolomics with other omics disciplines will not only enhance our understanding of metabolic pathways but also unlock novel biotechnological and pharmacological applications. The continued refinement of high-throughput techniques will be instrumental in uncovering the full potential of plant-based metabolites for agricultural and therapeutic innovations.

## 11 Conclusion

Fenugreek (*Trigonella foenum-graecum* L.), a traditionally valued crop, is now recognized as a multipurpose species with immense potential in nutraceutical, pharmaceutical, and agricultural sectors. Over the years, substantial progress has been made in its genetic improvement through a combination of conventional breeding strategies and modern biotechnological interventions. Mutation breeding has played a pivotal role in expanding the genetic base of fenugreek, enabling the development of early-maturing, high-yielding, and metabolite-rich genotypes.

The application of diverse molecular markers—such as RAPD, AFLP, ISSR, iPBS, and SNPs—has provided deeper insights into the genetic variability and population structure of fenugreek germplasm, which are essential for trait-based selection and cultivar development. These marker systems, combined with phenotypic and biochemical analyses, have improved the accuracy and efficiency of breeding programs.

In parallel, omics technologies have offered a broader understanding of the molecular mechanisms governing trait expression. Transcriptomic studies have unraveled gene networks associated with diosgenin biosynthesis and stress response, while proteomic and metabolomic analyses have shed light on the dynamic changes in protein expression and metabolite accumulation under variable conditions. This systems-level approach has revealed regulatory pathways that can be strategically manipulated for enhanced secondary metabolite production and stress resilience.

Biotechnological advancements, particularly *in vitro* systems such as callus, suspension, and hairy root cultures, have further supported the production of pharmaceutically important compounds like trigonelline and steroidal saponins. Although challenges persist in establishing efficient protoplast regeneration and complete *in vitro* plant development, recent studies indicate promising leads. With emerging genome editing technologies such as CRISPR/Cas systems, there is growing potential to overcome existing limitations and introduce targeted modifications in key metabolic pathways.

However, several areas remain underexplored. The absence of a reference genome continues to hinder deeper genomic studies and precision gene editing. Moreover, molecular mechanisms underlying biotic stress resistance and root-associated traits need further investigation. Integrating omics data with phenotypic observations, environmental adaptability, and predictive modeling will be crucial in bridging these gaps.

To fully realize the potential of fenugreek as a functional food and medicinal crop, future research must adopt an integrated and interdisciplinary approach. Strengthening the synergy between conventional breeding, omics sciences, and scalable *in vitro* technologies will not only accelerate the development of superior cultivars but also support the growing demand for plant-based therapeutics and climate-smart agriculture.
